# Supplementary material for: Paternal SARS-CoV-2 infection impacts sperm small noncoding RNAs and increases anxiety in offspring in a sex-dependent manner
Source: Nat Commun. 2025 Oct 11;16:9045. doi: 10.1038/s41467-025-64473-0 (PMC12515249; doi:10.1038/s41467-025-64473-0)
Supplement: Supplementary file 1 — Supplementary Information File [file 41467_2025_64473_MOESM1_ESM.pdf]

Supplementary Figures

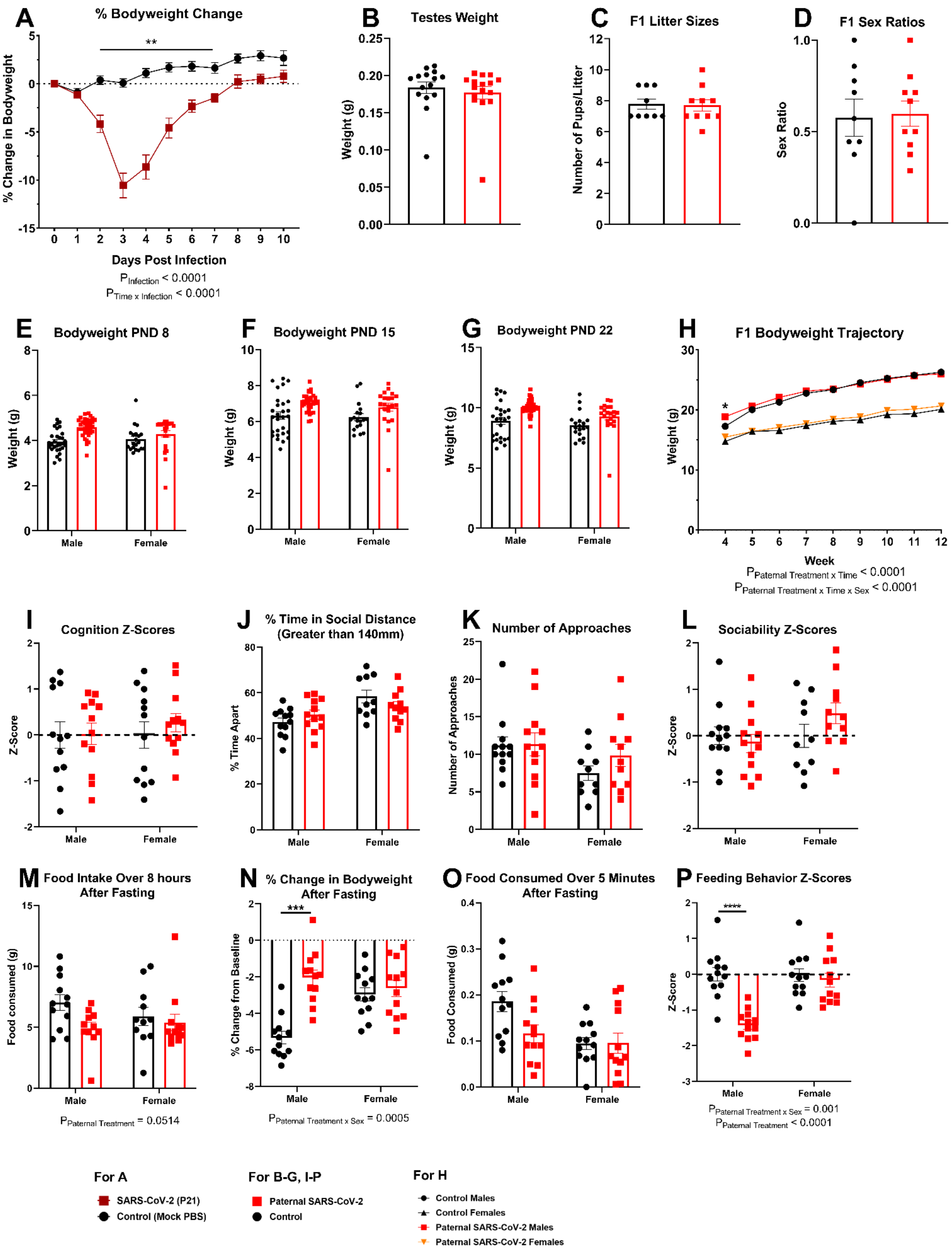

Supplementary Figure 1. SARS-CoV-2 infection in male mice affects the bodyweight trajectory of adult offspring without changing litter characteristics. SARS-CoV-2 infection significantly affects A) % bodyweight change over 10 days post-infection (n=10), without affecting B) testes weight (n=15 CON, n=16 SARS), C) F1 litter sizes (n=9 CON, n=10 SARS litters per group), and D) F1 litter sex ratios (n=9 CON, n=10 SARS litters per group)(calculated as a proportion of male pups in each litter). Paternal SARS-CoV-2 infection does not significantly change F1 bodyweight at E) PND 8 (n=29 CON M, n=20 CON F, n=36 SARS M, n=24 SARS F), F) PND 15 (n=28 CON M, n=18 CON F, n=36 SARS M, n=21 SARS F) and G) PND 22 (n=26 CON M, n=19 CON F, n=36 SARS M, n=21 SARS F) after correcting for litter effects. Paternal SARS-CoV-2 infection significantly alters the H) bodyweight trajectory in adult offspring (n=12). Paternal SARS-CoV-2 has no significant impact on I) overall cognition behavioral z-scores (n=12), J) % time spent greater than 140mm apart from the guest mouse over 10 minutes (n=12 CON M, n=10 CON F, n=12 P.SARS M, n=11 P.SARS F), K) number of approaches to the guest mouse made over 10 minutes (n=12 CON M, n=10 CON F, n=12 P.SARS M, n=11 P.SARS F), and L) overall sociability behavioral z-scores (n=12 CON M, n=10 CON F, n=12 P.SARS M, n=11 P.SARS F). There was a trend towards reduced M) Food consumed over 8 hours after an 18-hour fasting period (n=11 CON M, n=11 CON F, n=12 P.SARS M, n=11 P.SARS F). Paternal SARS-CoV-2 significantly alters N) % bodyweight changed after a 24-hour fasting period in male offspring (n=12)(P<0.0001, general linear model with Bonferroni correction), without changing O) food consumed in a 5-minute period after a 24-hour fasting period for F1 offspring (n=12). Paternal SARS-CoV-2 significantly affects P) overall feeding behavioral z-scores (n=12)((P<0.0001, general linear model with Bonferroni correction). Data presented as mean ± SEM. A two-way ANOVA with Sidak's multiple comparisons test was used to analyse A. A Mann-Whitney U-test was used to analyse B-D. Linear mixed models were used to analyse E-P with *post-hoc* analyses where appropriate (Bonferroni-Holm corrected). Each n number refers to the number of individual animals per group. $P_{\text{Paternal Treatment}}$ = main effect of paternal treatment or $P_{\text{Paternal Treatment x time}}$ = interaction of paternal treatment by time. *P<0.05, **P < 0.01, ***P<0.001, ****P<0.0001.

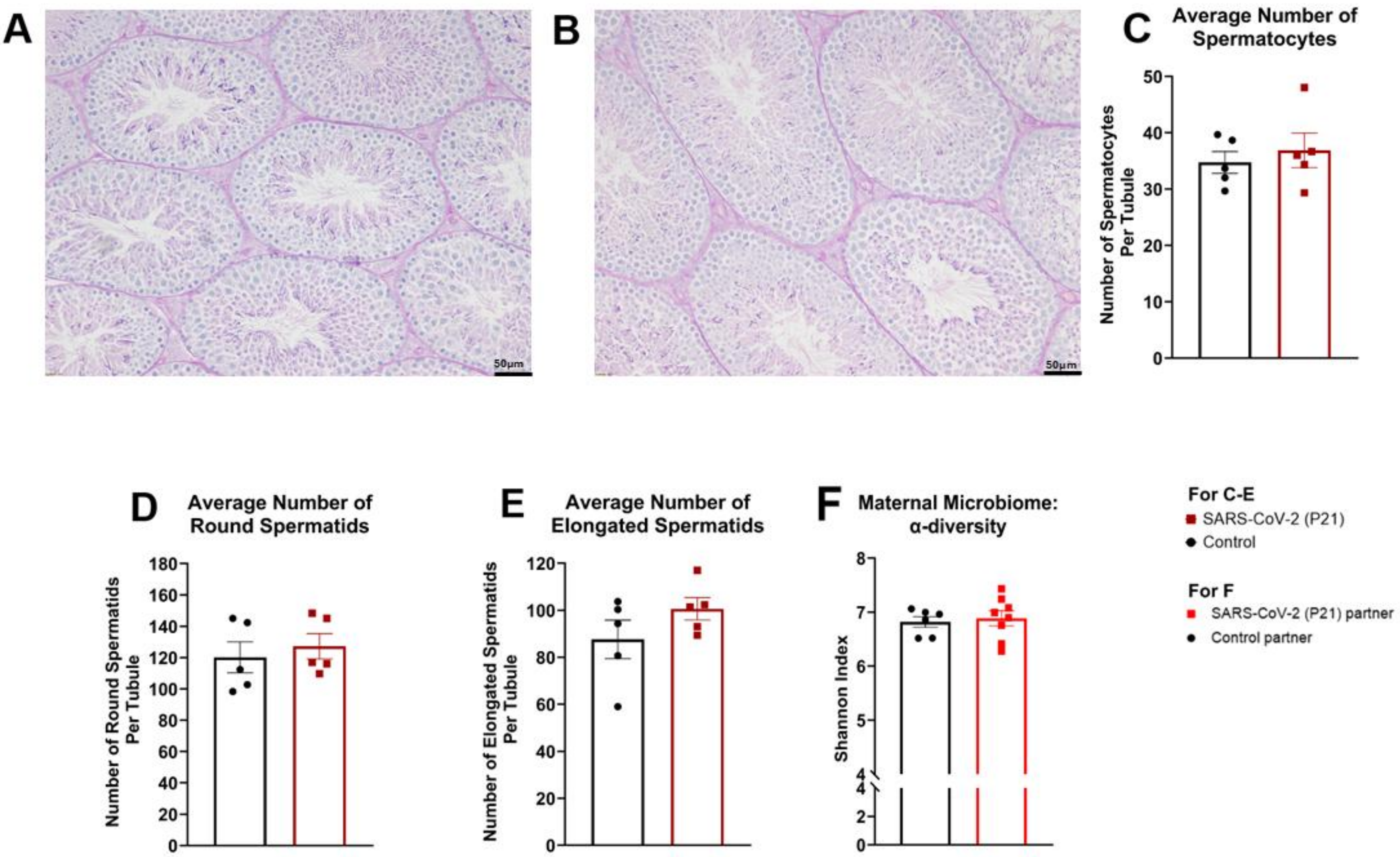

Supplementary Figure 2. Representative images of a periodic acid Schiff and haemotoxylin stained A) mock infected (control) mouse testis section (5µm), and B) SARS-CoV-2 infected mouse testis section (5µm). C) Average number of spermatocytes/tubule for each mouse (across 3 tubules per mouse: n=5 mice per group), D) average number of round spermatids/tubule for each mouse (across 3 tubules per mouse: n=5 mice per group), and E) average number of elongated spermatids/tubule for each mouse (across 3 tubules per mouse: n=5 mice per group), F) α-diversity (Shannon index) of the fecal microbiome from naïve females (partners) bred with male mice previously exposed to either a mock infection (control) or SARS-CoV-2 (P21) infection (n=6 CON partner, n=8 SARS partner). A Mann-Whitney U-test was used to analyse C, D, and E. An unpaired two-tailed Student's t-test was used to analyse F. A Bars = 50µm.

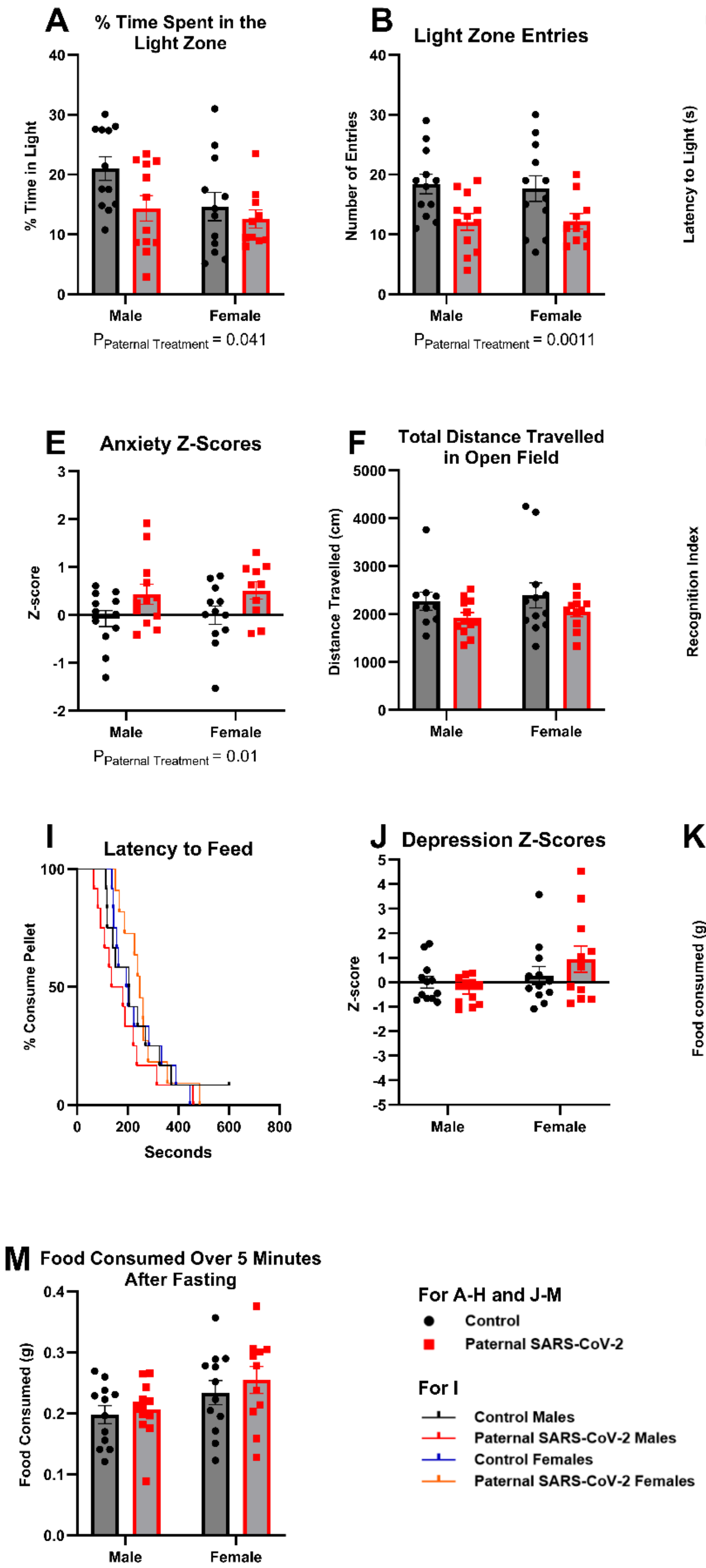
A % Time Spent in the Light Zone
% Time in Light
Male
Female
$P_{Paternal\ Treatment}$ = 0.041
B Light Zone Entries
Number of Entries
Male
Female
$P_{Paternal\ Treatment}$ = 0.0011
C Latency to Light Zone
Latency to Light (s)
Male
Female
$P_{Paternal\ Treatment}$ = 0.0487
D % Time Spent in the Centre
%Time in Centre
Male
Female
E Anxiety Z-Scores
Z-score
Male
Female
$P_{Paternal\ Treatment}$ = 0.01
F Total Distance Travelled in Open Field
Distance Travelled (cm)
Male
Female
G Recognition Index
Recognition Index
Male
Female
H % Sucrose Preference
% Sucrose Preference
Male
Female
I Latency to Feed
% Consume Pellet
Seconds
J Depression Z-Scores
Z-score
Male
Female
K Food consumed Over 8 Hours After Fasting
Food consumed (g)
Male
Female
L % Change in Bodyweight After Fasting
% Change from Baseline
Male
Female
$P_{Paternal\ Treatment}$ = 0.0489
M Food Consumed Over 5 Minutes After Fasting
Food Consumed (g)
Male
Female
For A-H and J-M
Control
Paternal SARS-CoV-2
For I
Control Males
Paternal SARS-CoV-2 Males
Control Females
Paternal SARS-CoV-2 Females

Supplementary Figure 3. Paternal SARS-CoV-2 infection significantly changes anxiety-like behavior in a separate cohort of F1 offspring. Paternal SARS-CoV-2 infection significantly decreases A) % time spent in the light zone of the light-dark box (n=12 CON M, n=12 CON F, n=12 SARS M, n=10 SARS F) and B) number of entries into the light zone (n=12 CON M, n=12 CON F, n=12 SARS M, n=10 SARS F) in a separate cohort of F1 offspring. Paternal SARS-CoV-2 infection significantly increases C) the latency to enter the light zone in the F1 offspring (n=12 CON M, n=12 CON F, n=12 SARS M, n=10 SARS F) without changing D) % time spent in the centre of the open-field (n=10 CON M, n=12 CON F, n=12 SARS M, n=11 SARS F) while increasing the E) overall anxiety behavioral z-scores (n=12 CON M, n=12 CON F, n=12 SARS M, n=11 SARS F) for F1 offspring. Paternal SARS-CoV-2 has no significant effects on F) the total distance travelled in the open-field (n=10 CON M, n=12 CON F, n=12 SARS M, n=11 SARS F), G) the recognition index in trial 2 of the novel-object recognition test (n=12 CON M, n=12 CON F, n=12 SARS M, n=11 SARS F), H) % preference for sucrose in the sucrose preference test (n=12 CON M, n=10 CON F, n=10 SARS M, n=11 SARS F), and I) latency to feed in the novelty-suppressed feeding test for F1 offspring (n=12 CON M, n=12 CON F, n=12 SARS M, n=11 SARS F). Paternal SARS-CoV-2 does not significantly alter J) overall depression behavioral z-scores (n=12 CON M, n=12 CON F, n=12 SARS M, n=11 SARS F), nor does it change K) food consumed over 8-hours after an 18-hour fasting period (n=12 CON M, n=10 CON F, n=10 SARS M, n=11 SARS F). Paternal SARS-CoV-2 significantly alters L) % bodyweight changed after a 24-hour fasting period (n=12 CON M, n=12 CON F, n=12 SARS M, n=11 SARS F) but does not significantly change M) food consumed over 5 minutes after a 24-hour fasting period (n=12 CON M, n=12 CON F, n=12 SARS M, n=11 SARS F). Data presented as mean ± SEM. General linear models and linear mixed models were used with *post-hoc* analyses where appropriate (Bonferroni-Holm corrected) except for I. Cox regression with proportional hazards was used to analyse I. Each n number refers to the number of individual animals per group. $P_{\text{Paternal Treatment}}$ = main effect of paternal treatment.

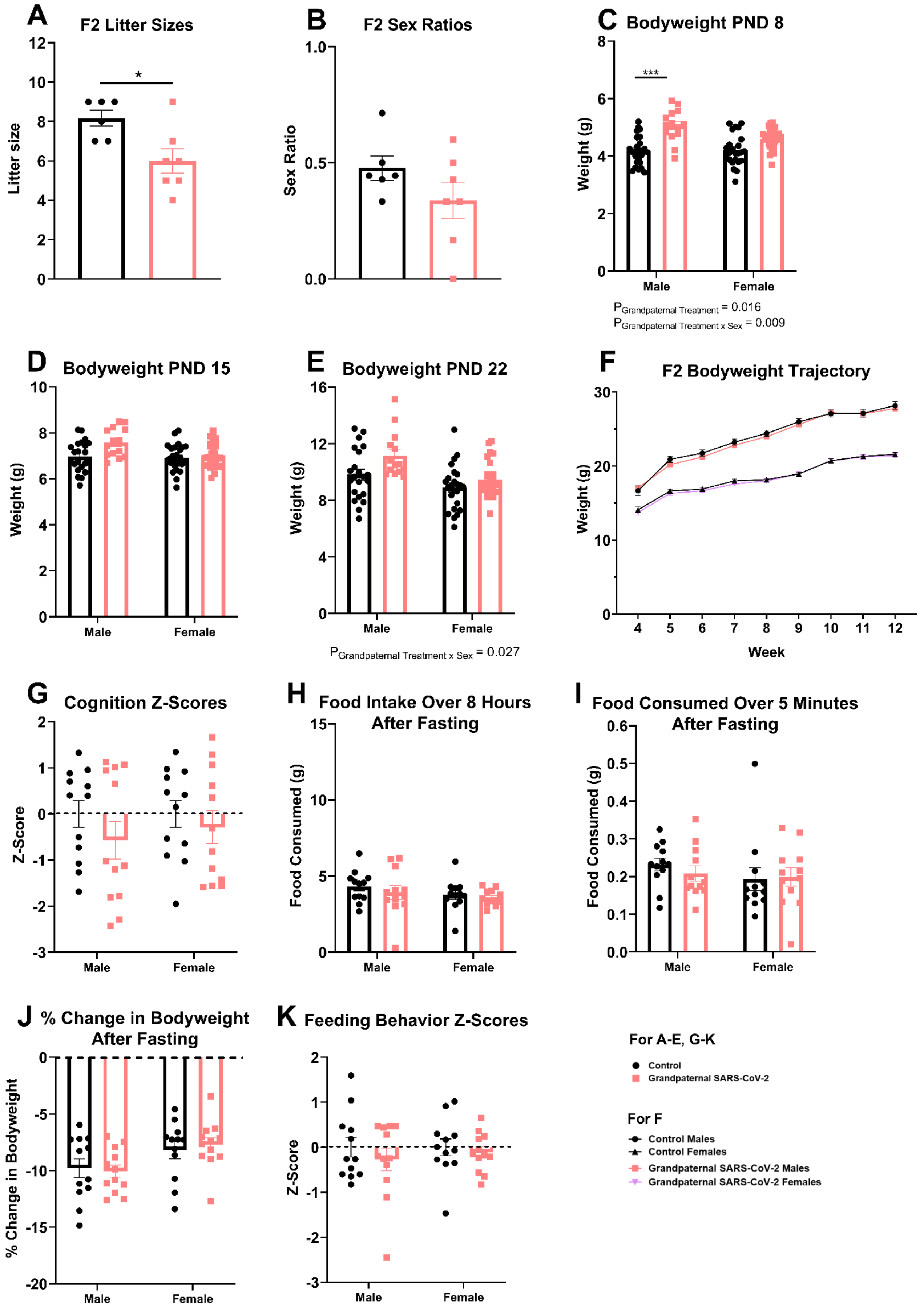

A F2 Litter Sizes
Litter size
*
B F2 Sex Ratios
Sex Ratio
C Bodyweight PND 8
Weight (g)
***
Male
Female
P Grandpaternal Treatment = 0.016
P Grandpaternal Treatment x Sex = 0.009
D Bodyweight PND 15
E Bodyweight PND 22
P Grandpaternal Treatment x Sex = 0.027
F F2 Bodyweight Trajectory
Week
G Cognition Z-Scores
Z-Score
H Food Intake Over 8 Hours After Fasting
Food Consumed (g)
I Food Consumed Over 5 Minutes After Fasting
J % Change in Bodyweight After Fasting
% Change in Bodyweight
K Feeding Behavior Z-Scores
For A-E, G-K
Control
Grandpaternal SARS-CoV-2
For F
Control Males
Control Females
Grandpaternal SARS-CoV-2 Males
Grandpaternal SARS-CoV-2 Females

Supplementary Figure 4. Grand-paternal SARS-CoV-2 infection significantly affects F2 litter sizes and pre-weaning bodyweight. Grand-paternal SARS-CoV-2 significantly reduces A) F2 litter sizes (n=6 CON, n=7 GP.SARS litters per group) without affecting B) F2 litter sex ratios (n=6 CON, n=7 GP.SARS litters per group)(calculated as a proportion of male pups in each litter). Grand-paternal SARS-CoV-2 significantly increases F2 bodyweight at C) PND 8 (n=25 CON M, n=24 CON F, n=14 GP.SARS M, n=28 GP.SARS F), without affecting D) PND 15 (n=22 CON M, n=24 CON F, n=14 GP.SARS M, n=28 GP.SARS F) and E) PND 22 (n=22 CON M, n=24 CON F, n=14 GP.SARS M, n=28 GP.SARS F) bodyweight after correcting for litter effects. Grand-paternal SARS-CoV-2 does not significantly affect the F) F2 bodyweight trajectory in adulthood (n=12), G) overall cognition behavioral z-scores (n=12), H) food consumed over 8 hours after an 18-hour fasting period (n=12), I) food consumed in a 5 minute period after a 24-hour fasting period for F1 offspring (n=12), J) % bodyweight changed after a 24-hour fasting period (n = 12), K) overall feeding behavioral z-scores (n=12). Data presented as mean ± SEM. A Mann-Whitney U-test was used to analyse A and B. Linear mixed models were used with *post-hoc* analyses where appropriate (Bonferroni-Holm corrected) for C-F. Each n number refers to the number of individual animals per group. *P < 0.05, ***P < 0.001.

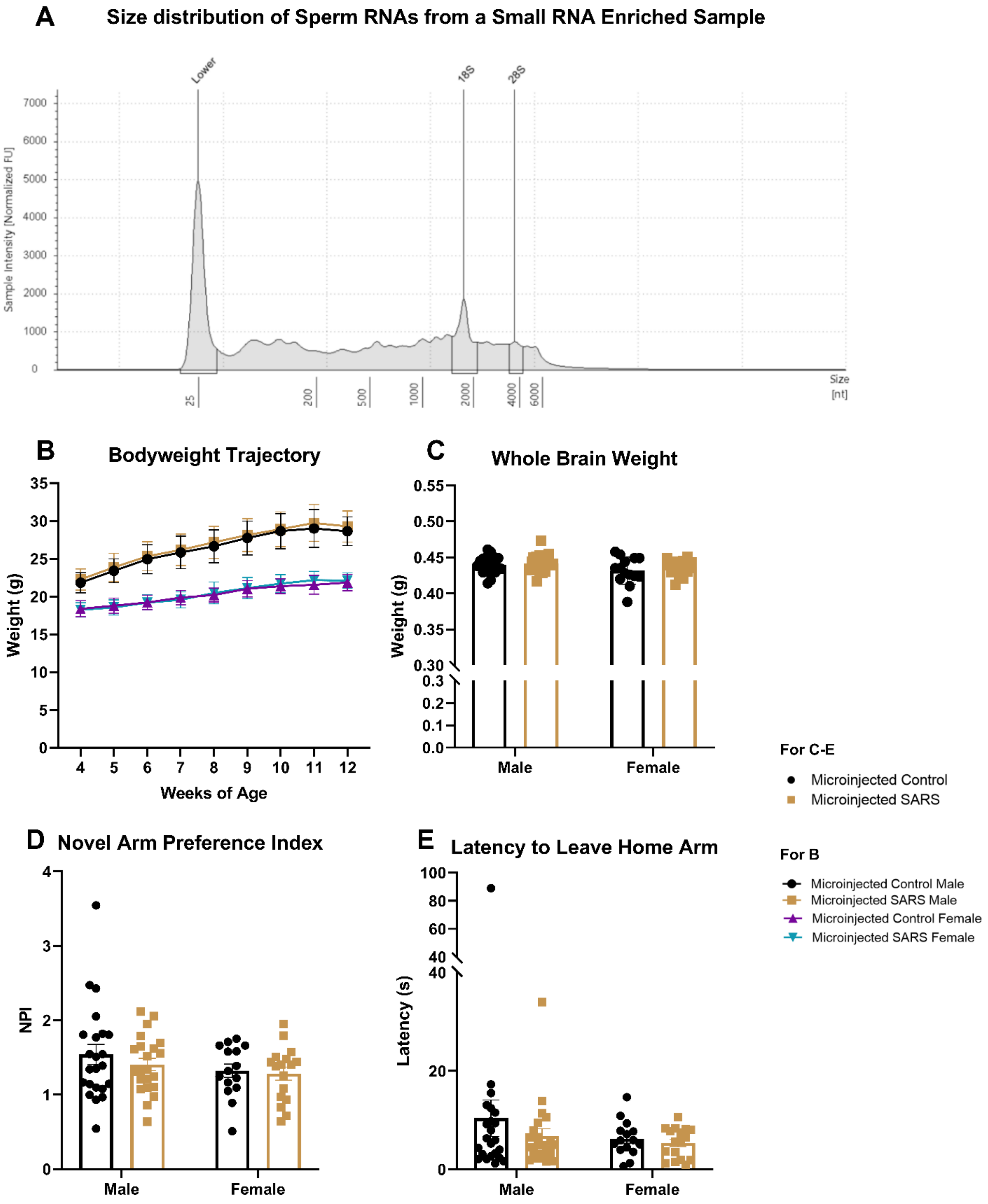

A
Size distribution of Sperm RNAs from a Small RNA Enriched Sample
Lower
18S
28S
Sample Intensity [Normalized FU]
7000
6000
5000
4000
3000
2000
1000
0
25
200
500
1000
2000
4000
6000
Size [nt]
B
Bodyweight Trajectory
Weight (g)
35
30
25
20
15
10
5
0
4 5 6 7 8 9 10 11 12
Weeks of Age
C
Whole Brain Weight
Weight (g)
0.55
0.50
0.45
0.40
0.35
0.30
0.3
0.2
0.1
0.0
Male
Female
For C-E
Microinjected Control
Microinjected SARS
For B
Microinjected Control Male
Microinjected SARS Male
Microinjected Control Female
Microinjected SARS Female
D
Novel Arm Preference Index
NPI
4
3
2
1
0
Male
Female
E
Latency to Leave Home Arm
Latency (s)
100
80
60
40
40
20
0
Male
Female

Supplementary Figure 5. A) Size distribution of RNAs found in a pooled control sperm RNA sample after the small RNA enrichment protocol. Microinjected-SARS mice show no changes in B) their bodyweight trajectory from 4 weeks old until 12 weeks old (n=25 MCON M, n=16 MCON F, n=22 MSARS M, n=19 MSARS F), C) whole brain weight (n=20 MCON M, n=14 MCON F, n=20 MSARS M, n=17 MSARS F), D) novel arm preference in the Y-maze (n=23 MCON M, n=15 MCON F, n=22 MSARS M, n=17 MSARS F), and E) latency to leave the home arm of the Y-maze in trial 2 (n=23 MCON M, n=15 MCON F, n=22 MSARS M, n=17 MSARS F). Data presented as mean ± SEM. General linear models were used with *post-hoc* analyses where appropriate (Bonferroni-Holm corrected). Each n number refers to the number of individual animals per group.

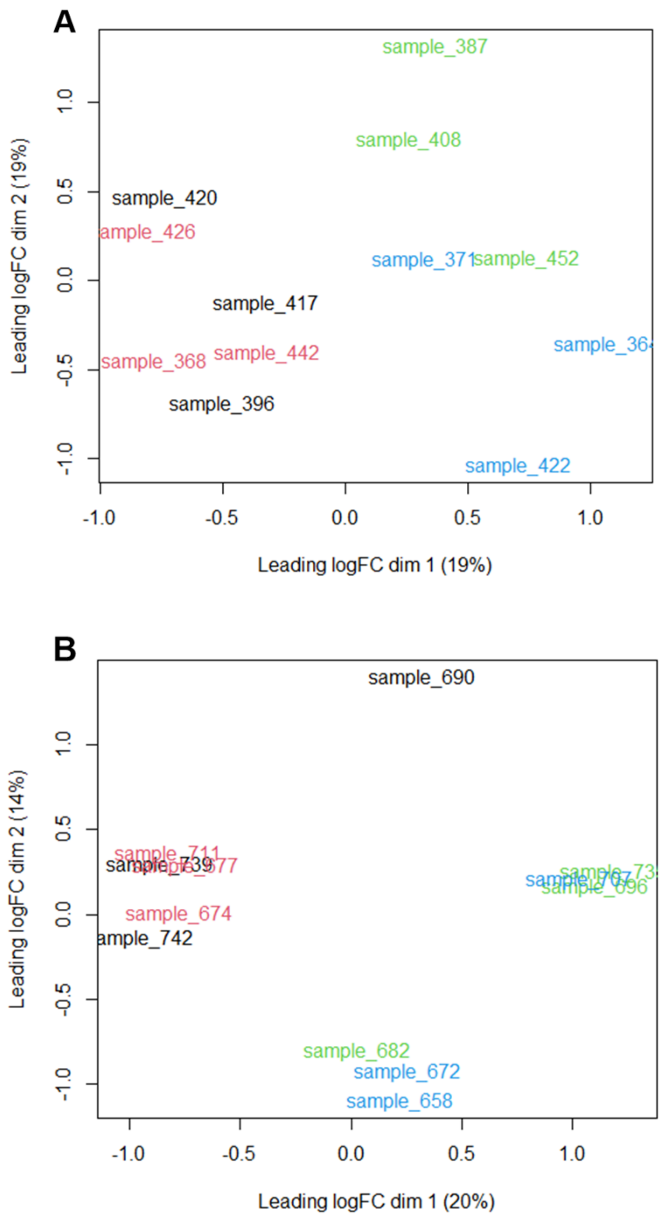

Supplementary Figure 6. A) Multidimensional scaling plot showing the relationship between F1 hippocampus mRNA samples according to biological sex and paternal treatment (red= F1 males from mock infected sires, black= F1 males from SARS-CoV-2 infected sires, blue= F1 females from mock infected sires, green= F1 females from SARS-CoV-2 infected sires), B) Multidimensional scaling plot showing the relationship between RNA-microinjected hippocampus mRNA samples according to biological sex and paternal treatment (red= males microinjected with sperm RNA from mock infected sires, black= males microinjected with sperm RNA from SARS-CoV-2 infected sires, blue= females microinjected with sperm RNA from mock infected sires, green= females microinjected with sperm RNA from SARS-CoV-2 infected sires).

Supplementary Table 1. piRNA cluster genomic information (top 50 clusters)

Table. Genomic information of top50 differentially expressed piRNA clusters

Clusters	Chromosome	Start	End	FDR	Average number of piRNA hits	Gene
cluster_75	chr14	65971728	65981528	2.12E-05	99.56	Clu
cluster_272	chr14	65971766	65977032	2.12E-05	59.78	Clu
cluster_678	chr14	65973148	65982034	2.12E-05	92.11	Clu
cluster_1675	chr14	65971193	65981513	2.12E-05	100.11	Clu
cluster_697	chr17	40291795	40310011	0.141562071	334.78	Crisp1
cluster_1248	chr17	40290490	40312969	0.141562071	338.89	Crisp1
cluster_95	chr17	40305059	40312420	0.280212635	145.89	Crisp1
cluster_196	chr9	109816652	109826616	0.280212635	141.89	Spink8
cluster_295	chr17	40305063	40308973	0.280212635	141.89	Crisp1
cluster_497	chr17	40303051	40309865	0.280212635	146.67	Crisp1
cluster_614	chr9	109819224	109828719	0.280212635	127	3000002C10Rik, Spink8
cluster_895	chr17	40305063	40309559	0.280212635	143.11	Crisp1
cluster_1068	chr17	40305073	40309794	0.280212635	143.22	Crisp1
cluster_1466	chr17	40301289	40311540	0.280212635	166.11	Crisp1
cluster_1695	chr17	40303864	40308972	0.280212635	144	Crisp1
cluster_1692	chr17	34996792	35000999	0.374017484	20.89	Vars
cluster_776	chr7	131316122	131320241	0.387726931	38.56	Cuzd1, Fgfr2
cluster_1783	chr7	131316111	131320746	0.387726931	38.89	Cuzd1, Fgfr2
cluster_4	chr4	82727	88481	0.674731682	186	Unknown
cluster_7	chr4	136421	142882	0.674731682	80.89	Unknown
cluster_9	chr1	24611586	24616128	0.674731682	252.33	Gm28439, Gm10222, Gm28438, Gm28437, Gm10925, Gm28661, Gm29216
cluster_19	chr10	4353151	4359323	0.674731682	357	Akap12
cluster_27	chr10	80052449	80057309	0.674731682	45.33	Gpx4
cluster_28	chr10	80332676	80336405	0.674731682	72.67	Reep6
cluster_29	chr10	80636964	80641579	0.674731682	66.67	Csnk1g2
cluster_30	chr10	85744101	85769872	0.674731682	1250.89	Unknown
cluster_32	chr10	85850066	85854295	0.674731682	53.78	Unknown
cluster_34	chr10	86587093	86627997	0.674731682	935.22	Unknown
cluster_35	chr10	86691051	86696806	0.674731682	51.22	Gm15344, 1810014B01Rik, Hsp90b1
cluster_41	chr11	50940868	50943578	0.674731682	140.78	4933414I15Rik
cluster_46	chr11	95828444	95832116	0.674731682	57.44	Abi3, Phospho1, Zfp652
cluster_48	chr11	103423295	103451991	0.674731682	1493	Lrrc37a
cluster_49	chr11	105933480	105937956	0.674731682	28.22	Cyb561
cluster_50	chr11	106782149	106786986	0.674731682	43.56	Ddx5, Mir3064, Gm25994
cluster_54	chr12	103672170	103676802	0.674731682	46	Serpina16
cluster_55	chr12	103689527	103695420	0.674731682	85.44	Serpina1f
cluster_60	chr13	50195080	50201301	0.674731682	113.78	Unknown
cluster_61	chr13	50300944	50308912	0.674731682	143.44	Unknown
cluster_66	chr13	53397907	53403534	0.674731682	334.22	Gm2762
cluster_68	chr14	24082139	24121763	0.674731682	3164.89	Mir7210
cluster_71	chr14	24424792	24430520	0.674731682	104.11	Unknown
cluster_72	chr14	31010896	31020657	0.674731682	163.22	Glt8d1, Snord69, Gm24916, Snord19, Gnl3, Pbrm1
cluster_76	chr14	73362384	73366980	0.674731682	148.44	Itm2b
cluster_78	chr15	59246219	59298559	0.674731682	3619.33	Unknown
cluster_80	chr15	74634180	74648696	0.674731682	2025.44	Mroh4
cluster_81	chr15	74653170	74661746	0.674731682	170	Unknown
cluster_84	chr15	78651111	78670843	0.674731682	1229.67	Elfn2
cluster_87	chr15	83353728	83360255	0.674731682	461.67	1700001L05Rik
cluster_88	chr16	20677035	20689010	0.674731682	170.67	Snord66, Eif4g1
cluster_90	chr17	23815247	23823607	0.674731682	68.89	Mir5125, Srrm2
